# Staying Alive or Going to Die During Terminal Senescence—An Enigma Surrounding Yield Stability

**DOI:** 10.3389/fpls.2015.01070

**Published:** 2015-11-30

**Authors:** Krishna S. V. Jagadish, Polavarapu B. Kavi Kishor, Rajeev N. Bahuguna, Nicolaus von Wirén, Nese Sreenivasulu

**Affiliations:** ^1^International Rice Research Institute, Metro Manila, Philippines; ^2^Department of Genetics, Osmania University, Hyderabad, India; ^3^Leibniz Institute of Plant Genetics and Crop Plant Research, Gatersleben, Germany

**Keywords:** carbohydrate remobilization, cereals, drought stress, heat stress, photosynthesis, senescence, stay-green

## Abstract

Breeding programs with the aim to enhance yield productivity under abiotic stress conditions during the reproductive stage of crops is a top priority in the era of climate change. However, the choice of exploring stay-green or senescence phenotypes, which represent an opposing physiological bearing, are explored in cereal breeding programs for enhanced yield stability to a different extent. Thus, the consideration of stay-green or senescence phenotypes is still an ongoing debate and has not been comprehensively addressed. In this review, we provide arguments for designing a target phenotype to mitigate abiotic stresses during pre- and post-anthesis in cereals with a focus on hormonal balances regulating stay-green phenotype versus remobilization. The two major hypothesis for grain yield improvement are (i) the importance of the stay-green trait to elevate grain number under pre-anthesis and anthesis stress and (ii) fine tuning the regulatory and molecular physiological mechanisms to accelerate nutrient remobilization to optimize grain quality and seed weight under post-anthesis stress. We highlight why a cautious balance in the phenotype design is essential. While stay-green phenotypes promise to be ideal for developing stress-tolerant lines during pre-anthesis and fertilization to enhance grain number and yield *per se*, fine-tuning efficient remobilizing behavior during seed filling might optimize grain weight, grain quality and nutrient efficiency. The proposed model provides novel and focused directions for cereal stress breeding programs to ensure better seed-set and efficient grain-filling in cereals under terminal drought and heat stress exposure.

## Introduction

The World’s major cereal crops, i.e., rice (*Oryza sativa*), wheat (*Triticum aestivum*), and maize (*Zea mays*) account for more than 70% of the total production, provides a major share of the world’s caloric demand to help sustain global food security (Swaminathan, 2010). Global grain yield productivity of the above cereals more than doubled during the past six decades, but the rate of increase has slowed down considerably due to lack of genetic gain (Swaminathan, 2010; McKersie, 2015). The pressure to further improve germplasm is enhanced with the predicted increase in intensity and magnitude of drought and heat stress events under changing climate (Sreenivasulu et al., 2007; Dolferus, 2014). In particular over the last decade, erratic rainfall patterns and an enhanced frequency of heat waves have significantly reduced cereal production across the world and affected global agricultural production (Peng et al., 2004; Welch et al., 2010; Lobell and Gourdji, 2012). Using 9 years of satellite data, a +2°C scenario resulted in a 20% reduction in wheat yield losses along the Indo Gangetic Plain of Northern India due to significant acceleration of senescence and reduction in crop growing duration (Lobell and Gourdji, 2012; Lobell et al., 2012). Drought and heat stress occurrence, particularly during the terminal stages of plant growth cycle limits crop productivity world-wide by drastically decreasing grain number and altering seed filling events (Seiler et al., 2011, 2014; De Storme and Geelen, 2014; Raorane et al., 2015; Stratonovitch and Semenov, 2015). In addition, high temperature is also known to affect key grain quality traits, such as reduced head rice recovery, higher chalk percentage and impaired starch accumulation (Shi et al., 2013; Sreenivasulu et al., 2015). Hence, there is a need to breed varieties that can withstand such harsh environmental conditions providing options to extend cultivation to areas that are vulnerable to these stresses for sustaining food security under changing climate.

Drought and heat stress reduces photosynthesis and induces the onset of leaf senescence through the induction of a series of complex metabolic changes. Major physiological reprogramming events occur under severe stress exposure leading to chlorophyll degradation, production of reactive oxygen species (ROS), oxidation of proteins and lipids affecting source strength. ROS generated in chloroplasts and mitochondria during drought and heat may ultimately cause senescence of leaves and affect yield potential (Hui et al., 2012; Khanna-Chopra, 2012; Chen et al., 2013; Semenov et al., 2014). With the onset of senescence chloroplasts are dismantled and stromal enzymes are degraded leading to reduced photosynthesis, while mitochondria remain functional (Sakuraba et al., 2014). Rubisco degradation is required to meet the N demand of sink organs under senescence or accelerated senescence due to abiotic stresses (Gotz et al., 2007; Gomes de Oliveira Dal’Molin et al., 2015). Glutamine synthetase (GS) and Rubisco are the key enzymes for N and C assimilation, with the plastidial form of GS being degraded quicker than the cytosolic GS. A link has been proposed between chloroplasts, cytosol and vacuoles in the form of Rubisco vesicular bodies to be involved in the autophagocytosis of cytosolic and chloroplastic proteins (Prins et al., 2008). However, there is an ongoing debate on the location, the rate of Rubisco degradation, as well as the need for identifying autophagy-related genes involved in regulating Rubisco-containing bodies under natural and stress induced senescence.

Carbon and nitrogen are important resources which are liberated and recycled or remobilized for re-use in other growing parts of the plants during the senescence process. During stress and in naturally senescing leaves, sugars (glucose, fructose) are accumulated (Wingler et al., 2006). But, how sugars are accumulating despite a decline in photosynthesis in senescing leaves is still unknown. There are two possibilities for sugar accumulation under senescence: one is cleavage of starch, which was accumulated during pre-anthesis, and the other possibility is a higher availability of carbon from decreased amino acid synthesis (Jongebloed et al., 2004). In addition, low nitrogen and high light results in senescence of leaves and accumulation of sugars. These findings suggest that the balance between sugar and nitrogen during the sink/source transition of leaves can play a critical role in the induction of leaf senescence (Masclaux-Daubresse et al., 2014). *SAG12*, a senescence-specific gene was induced over 900-fold by glucose (Pourtau et al., 2006). In addition, trehalose 6-phosphate (considered to be a signal for high carbon availability) is required for the onset of leaf senescence associated with high carbon availability in *Arabidopsis* (Wingler et al., 2012). Though accumulation of hexoses in aging leaves is hypothesized to initiate or accelerate senescence, this alone may not trigger senescence, rather, it is the complex network of other metabolites (nitrogen) and environmental factors.

While nitrogen deficiency induces leaf senescence (nitrogen deficiency induced senescence, NDI senescence) and increases N recycling and remobilization, higher or optimal N concentrations promote leaf growth and greenness (Diaz et al., 2008). Therefore, improving N use efficiency (NUE) of crop plants is important under water deficit conditions. It is a well known fact that water deficit enhanced senescence in wheat by accelerating loss of leaf nitrogen and leaf chlorophyll and increasing lipid peroxidation and therefore phloem loading is crucial for efficient N remobilization. If N uptake during grain set is too low, the plant’s N demand cannot be met. This situation reduces cytokinin (CK) biosynthesis which induces leaf protein degradation. The amino acids that are released due to protein degradation are exported to the grains via the phloem. Up to 95% of seed proteins consist of amino acids that have been exported to the seed after protein degradation in rosette leaves (Fait et al., 2011). This illustrates that senescence is an important pre-requisite for remobilizing not just nitrogen but also other important nutrients. The chloroplast harbors a major pool of reduced leaf nitrogen. Hence, remobilization of nitrogen essentially includes the degradation of chloroplast proteins to various transportable forms of nitrogen (Hortensteiner and Feller, 2002). Different classes of proteases are activated during senescence to ensure that leaf proteins are degraded into amino acids which are eventually transported to the developing grains (Distelfeld et al., 2014). In conjunction, several findings have shown that sugars in combination with low nitrogen supply can induce senescence (Guiboileau et al., 2013; Avila-Ospina et al., 2015). Other internal cues that can induce senescence under natural or stress environments include hormones, transcription factors, the cellular redox state, and the sink strength which triggers nutrient remobilization.

A stay-green phenotype relies more on current photosynthesis and retains more functional leaf chlorophyll that enables them to synthesize carbohydrates and to provide assimilates during anthesis as well during seed development. The delayed onset or slower rate of senescence has been described to be advantageous in *Sorghum bicolor* (Borrell et al., 2014), *Triticum aestivum* (Spano et al., 2003), *Hordeum vulgare* (Seiler et al., 2014), *Pennisetum glaucum* (Sehgal et al., 2015), *Oryza sativa* (Fu et al., 2011b), and *Zea mays* (Cairns et al., 2012; Almeida et al., 2014), showing positive correlations between water use efficiency and final yields to combat terminal drought stress (Condon et al., 2004). The impact of a stay-green phenotype and the contribution of its photosynthetically active tissue in the spike under terminal drought is of high relevance in tribe Triticeae, but these mechanisms have not been explored (Tambussi et al., 2005; Raven and Griffiths, 2015). The lemma-derived awns usually grow long as bristle-like structures, possessing a smooth or rough (with minute barbed hooks) surface with stomata, which contribute toward production of photo-assimilates (Toriba and Hirano, 2014). Recent research re-emphasized the importance of spike photosynthesis and assimilate supply in optimizing grain yield under stressful conditions (Maydup et al., 2010; Reynolds et al., 2012; Zhou et al., 2014; Kohl et al., 2015).

On the contrary, in such adverse conditions, senescence triggers the remobilization of carbon and nitrogen from vegetative tissues (leaf canopy and stems) to the grains and accelerates the grain-filling rate. These events alter carbon and nitrogen metabolism and impair translocation mechanisms leading to source-sink disturbances which are regulated through the action of a complex web of hormones and a multilayered regulatory network of genes (Gregersen et al., 2013; Albacete et al., 2014; Thomas and Ougham, 2014). In principle, a combination of faster remobilization and enhanced grain-filling rate could outweigh the loss of reduced photosynthesis and the shortened grain-filling period which ultimately ensures improved grain weight and grain quality in cereals. In this scenario, unfavorably delayed leaf senescence is becoming a concern for poor grain filling in rice and wheat which leaves large amounts of water soluble carbohydrates unused in stems (Yang and Zhang, 2006; Li et al., 2015a; Liu et al., 2015; Zhang et al., 2015). The role of transporters activated during remobilization process plays an important role in fine tuning source-sink dynamics has been discussed elsewhere (Gregersen et al., 2008; Braun et al., 2014; Distelfeld et al., 2014) and therefore we will not discuss this topic in the present review.

From these circumstantial evidence, several intriguing questions arise: (i) Do we need to search for stay-green genotypes that are characterized by perseverance in photosynthesis during the time of seed set and fertilization and thus avoid seed abortion? (ii) Are target genotypes more advantageous when they possess a higher remobilization capacity during the later seed filling phase? and (iii) Is it possible to combine both phenomena to achieve drought and heat tolerant lines for anthesis and post-anthesis stress, and if so, what are the contributing molecular mechanisms?

Summarizing the outcome of the vast number of studies reporting on the complex link between yield potential and phenotypes related to staying alive (stay-green) or choosing cell death of the canopy (senescence) rather increases confusion than providing resolution (Gregersen et al., 2013). This is most likely due to the fact that major abiotic stresses induce imbalances in source (impairment in photosynthesis and/or induction of remobilization) and sink tissues (sterility and inefficiency in seed filling) to a different extent depending on the developmental stage (anthesis or post-anthesis). Also the intensity and duration of a stress and the ability to cope with stress based on the plasticity of a given genotype contribute to yield stability varies between cultivars and species. This review focuses on the progress achieved in addressing the mechanisms related to source-sink imbalance, weighs the key findings of stay-green and remobilization impact and provides future research direction in safeguarding yield stability and improving grain quality under pre- and post-anthesis drought and heat stress in cereals.

## Importance of the Stay-Green Trait to Elevate Grain Number Under Drought and Heat Stress

Stay-green phenotypes have been reported in several crops, like sorghum, barley, wheat, pearl millet, maize and rice, to confer crop yield improvement under terminal drought and heat stress (Spano et al., 2003; Fu et al., 2011b; Chen et al., 2013; Borrell et al., 2014; Seiler et al., 2014; Sehgal et al., 2015). Depending on the dynamics of accelerating or delaying senescence, “functional” stay-green types, characteristically possess active or extended photosynthesis under drought resulting in higher yield stability (Gregersen et al., 2013), mainly due to a reduction in reproductive organ sterility and improvement in seed set (Ji et al., 2010; Dolferus et al., 2011; Sreenivasulu and Schnurbusch, 2012; Dolferus, 2014). There are at least four types of stay-green phenotypes described depending on the dynamics of senescence (Thomas and Howarth, 2000; Thomas and Ougham, 2014). If senescence is initiated late and then proceeds at a normal rate, it is type A. In contrast, type B represents genotypes, in which senescence is initiated on schedule, but the rate of senescence proceeds comparatively slowly. In type C, though chlorophyll is retained indefinitely, senescence proceeds normally beneath the chlorophyll layer, and in type D, leaves remain stay-green with active photosynthesis and senescence onset is very slow (Thomas and Howarth, 2000). Stay-green mutants have been identified in a number of plant species (Table 1), and the pathway which distinguishes from “functional stay-green” with type C encompassing “cosmetic” mutants is being unraveled, where plants retain chlorophyll and remain green while their photosynthetic capacity is severely impaired (Sato et al., 2007; Hortensteiner, 2009; Zhou et al., 2011; Grassl et al., 2012; Luo et al., 2013; Thomas and Ougham, 2014). Stay-green mutants as *nyc1/nol* retained 10 times more chlorophyll in the seeds than the wild type due to lack of chlorophyll *b* reductase, seriously affecting seed development, maturation, viability and finally impairing their germination (Nakajima et al., 2012). Hence, distinguishing such cosmetic phenotypes from true stay-green lines requires physiological and biochemical markers to assess source-sink strength, when stress-tolerant lines are developed in breeding programs. Several attempts have been made to develop stay-green phenotypes in cereals using QTL mapping (Table 1) and molecular markers are derived (Almeida et al., 2014; Borrell et al., 2014; Rama Reddy et al., 2014; Sehgal et al., 2015). The significance of stay-green for drought tolerance is also evident from a number of transgenic plants over-accumulating CK relative to abscisic acid (ABA) through the overexpression of isopentenyl transferase (IPT; Werner et al., 2010) or fine-regulating the catabolism of ABA under terminal drought (Seiler et al., 2014). The enhanced drought tolerance of such transgenic plants was the result of an extended photosynthetic capacity and maintenance of green leaf area (Ma, 2008; Peleg et al., 2011; Merewitz et al., 2012; Seiler et al., 2014).

**TABLE 1:** **List of stay-green mutants and QTLs identified in different cereal species**.

**Species**	**Role of mutant or QTL**	**Reference**
*Oryza sativa* L.	*nyc1* mutant cosmetic, chlorophyll *b* reductase	Kusaba et al. (2007)
*Oryza sativa* L.	*sgr* mutant, cosmetic	Sato et al. (2009)
*Oryza sativa* L.	SNU-SG1, functional stay-green	You et al. (2007)
*Sorghum bicolor*	Stg1, 2, 3, 4, Stg A and B QTLs; Stay-green for higher tiller grain yield QL41, cosmetic	Thomas and Howarth, (2000)
*Triticum aestivum*	Functional stay-green QTLs	Spano et al. (2003), Naruoka et al. (2012)
*Triticum aestivum*	Functional stay-green XN901 QTL	Gong et al. (2005)
*Zea mays*	Functional stay-green FS854 QTL	Thomas and Howarth (2000), Almeida et al. (2014)

**FIGURE 1:**
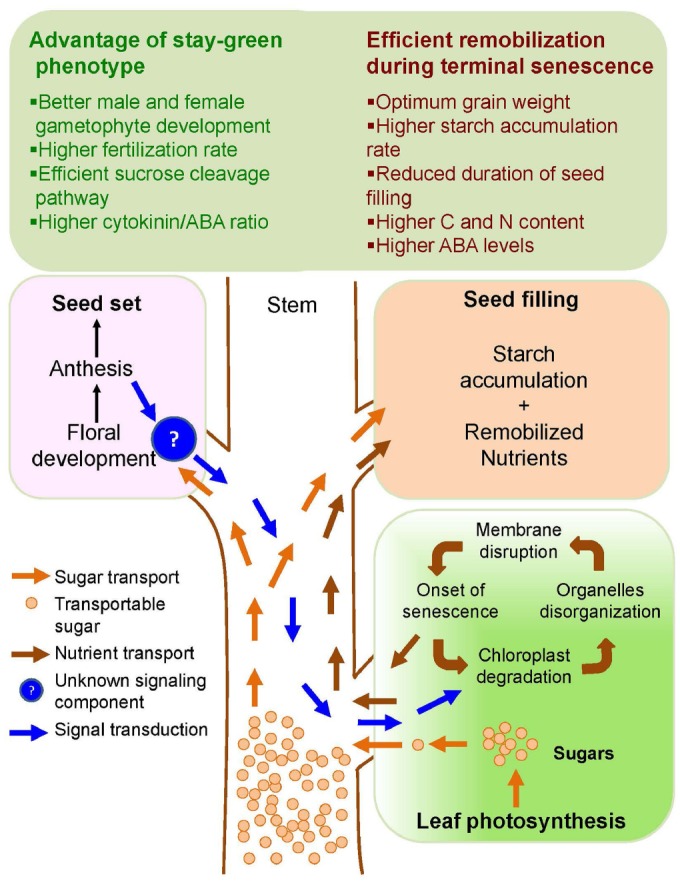
**Schematic diagram showing integrative effect of stay-green and terminal senescence traits in plants.** Extended stay-green trait provides sufficient photosynthate available as transportable sugar (orange arrows) for floral development and higher starch accumulation during grain filling. Conversely, initiation of terminal senescence after seed set provides additional nutrient supply (brown arrows) to the developing grains to improve optimum grain weight and quality. An unknown signaling component (?) from the floral organ (blue arrows) is thought to initiate the senescence process in leaves.

Several stress factors, in particular drought and heat stress, induced seed yield penalties that were conferred by different phenological alterations in the sink tissue, i.e., during the young microspore stage and subsequently during the anthesis and grain filling stage (Ji et al., 2010). Though crop yield reductions under abiotic stress are mainly a consequence of reduced grain number (Dolferus et al., 2011), the mechanisms of how stay-green phenotypes improve grain number under stress remain poorly understood. A highly flexible and dynamic adjustment due to premature abortion of developing florets under drought or heat stress exposure, can potentially occur throughout the spike development starting from floral meristem differentiation due to sugar starvation (Guo et al., 2015; Li et al., 2015b), which could be averted by reducing ABA level and by elevating brassinosteroids (Ji et al., 2011; Zhu et al., 2015). Under high night temperatures exposure from panicle initiation onwards, a significant floret degeneration was attributed to a competition for assimilates between the growing stem and developing ear (Shi et al., 2013). Moreover, a significant drop in peduncle elongation further increased floret sterility under drought stress mainly due to a shortage of assimilates and competition between stem and ear for the depleted assimilate pool (Jagadish et al., 2010). Two key processes that could determine the viability of reproductive organs and thereby grain numbers are the availability of sufficient amounts of sugars through maintained photosynthesis and efficient sucrose cleavage pathway to channel gradients of hexoses to the developing reproductive tissues (Ji et al., 2010; Dolferus et al., 2011; Suneja et al., 2015). Hence, mechanisms that impact grain numbers through key physiological events including altered male and female gametophyte development, pollen and ovule viability, fertilization events and optimum seed filling are key drivers for maintaining yield under stress (Figure 1).

Heat stress (39°C) during the sensitive microspore stage in rice, led to a failure of tapetal degeneration affecting pollen grain wall composition and causing poor adherence of pollen onto the stigma (Endo et al., 2009). Similar phenomena were observed during barley meiosis leading to aborted tapetal and pollen mother cell development leading to dramatic transcriptome reprogramming (Oshino et al., 2007). Physiological mechanisms including tapetal dysfunction, microspore collapse, loss of pollen and ovule viability, anther indehiscence, lack of pollen adhesion on stigma and poor pollen germination and fertilization are a series of cascading effects that may follow during anthesis and post-anthesis stress. In the male gametophyte development, the meiotic cell division is affected by stress factors, thereby disturbing the events connected to DNA replication followed by two rounds of chromosome segregation (MI and MII), crossover formation and recombination. The overall rate of recombination has been found to increase substantially mostly due to elevated ABA concentrations under stress. ABA positively regulates meiotic recombination 11 (MRE 11), an exo/endonuclease, leads to enhanced crossover (Prado and Aguilera, 2003) and also ABA, which is known to downregulate RAD51 causing chromosome fragmentation (Dray et al., 2006). The plasmadesmatal connections become undetectable and apoplastic transport is impaired under stress leading to a disturbance in sugar transport from the tetrad to the mature pollen stage, which is also known to be influenced by ABA (Oliver et al., 2007; Nguyen et al., 2010). The active portrayal of the tapetum and the functional role of invertases in determining pollen viability and seed set is well documented (Proels et al., 2003; Oliver et al., 2007; Ji et al., 2011). Key tapetal cell wall invertase genes *IVR1* in wheat and *OsINV4* in rice repressed by water stress led to increased starch accumulation on anther walls (Koonjul et al., 2005; Oliver et al., 2007). The reduced cell wall invertase under short and long term heat stress during microspore meiosis led to irreversibly altered carbohydrate metabolism inducing starch deficiency and pollen abortion in rice, sorghum, and tomato (Jain et al., 2007, 2010; Li et al., 2012a, 2015b). With heat stress exposure, *Mha1* (plasma membrane H^+^-ATPase), *SUT3* and *MST7* (sucrose and monosaccharide transporter proteins), transcripts were highly abundant despite poor pollen viability and low seed set with an irreversible decline in ICW (*SbIncw1*) in sorghum microspores (Jain et al., 2010). Similarly, drought-stressed rice anthers accumulated high amounts of sucrose due to regulated expression of sucrose (*OsSUT5*) and monosaccharide transporter (*OsMTS7*) with repressed expression of cell wall invertase (*OsCIN4*, Nguyen et al., 2010). Further, the spatial expression of *OsSUT5* and *OsMTS7* were mainly detected in young microspores, the tapetum and the anther middle layer and thereby indicated no restriction of sugar flow from anther walls to the developing microspores with invertases being the major bottleneck. Looking at a wider genetic pool and contrasting entries, drought-tolerant wheat germplasm maintained carbohydrate accumulation in the reproductive organs throughout stress duration by virtue of their ability to control and maintain sink strength and carbohydrate supply to the anthers (Ji et al., 2010). Invertase *IVR1* located in the wheat anther tapetum and around the vascular bundles was not repressed in drought-tolerant lines but strongly inhibited in the susceptible entries (Ji et al., 2010). Similarly, expression of the fructan biosynthesis genes *I-SST* and *6-FST* was reduced only in the susceptible wheat germplasm (Ji et al., 2010).

The regulatory pathway that controls anther sink strength and cell wall invertase activity remains elusive (Dolferus et al., 2011). Moreover, pollen sterility can be induced without reduction in spikelet water potential, and the ABA signal from the leaves is considered to trigger pollen collapse (Oliver et al., 2007). On the other hand, during the extremely metabolically active young microspore stage in the rice anther tapetum, mitochondrial numbers have been shown to increase by 20- to 40-fold (Dunwell and Sunderland, 1976; Mamun et al., 2005), to meet the high energy demand (Dolferus et al., 2011). In addition, poor or disturbed mitochondrial metabolism in the anther tapetum led to premature tapetal death, resulting in pollen abortion (Liu and Fan, 2013). Hence, in anthers a continuous supply of sugars is essential to maintain the pollen and ovule viability by meeting their hugely enhanced energy demand, and hence only a functional stay-green phenotype safeguards reproductive organ energy demand under stress.

Ovaries of cereals are normally loaded with glucose and starch on the day of pollination under control conditions (McLaughlin and Boyer, 2004). The sink strength of the ovary increases and gets to the highest point after fertilization and during grain filling where ovary growth cessation has been correlated with reduced sugars and depletion of starch (Zinselmeier et al., 1995). When the delivery of photosynthates is curtailed at low water potentials during drought, enzymes that metabolize sucrose, in particular the cell wall and soluble invertases loose activity (Zinselmeier et al., 1995; McLaughlin and Boyer, 2004). Under these conditions, previously accumulated starch is consumed through activation of amylases (Ruan, 2014), resulting often in seed abortion. The starch depletion in the wheat ovary is reversible, while that of the pollen is irreversible (Ji et al., 2010). The placento-chalazal cell wall invertase activity in ovules of open-pollinating maize is substantially reduced under drought stress restricting sugar delivery to the pedicel phloem. This resulted in a decrease of the sugar gradient between the pedicel and the nucellus surrounding the ovary sac and in ovary abortion (McLaughlin and Boyer, 2004; Makela et al., 2005). An initial down-regulation of invertases (*Incw 1-4*, *Ivr1-2*) and sucrose synthases (*SS1*, *SS2*) in maize ovaries following drought stress triggered a ribosomal inactivating protein (*RIP2*) and phospholipase D (*PLD1*), an indicator of membrane damage and irreversible loss of ovary viability (Andersen et al., 2002; McLaughlin and Boyer, 2004; Boyer and McLaughlin, 2007). ABA is known to repress cell wall invertases. The overexpression of ABA catabolism gene ABA-8′-hydroxylase maintained sink strength in wheat under cold stress (Ji et al., 2011). Increased accumulation of ABA in ovaries and reduced endogenous auxin levels in the anthers resulted in female flower and anther sterility, respectively (Vriezen et al., 2008; McAtee et al., 2013). The implications discussed in improving seed set lies in improved carbohydrate availability, transport and utilization. Hence, a stay-green phenotype meets the huge energy demand mentioned above and allows to reduce the heat- and drought-induced pre-anthesis at anthesis, fertilization and early embryo formation losses in grain number.

## Importance of Accelerated Nutrient Remobilization as a Trait to Optimize Grain Quality and Seed Weight Under Post-Anthesis Drought and Heat Stress

An alternative source of assimilates are pre-anthesis stem reserves in the form of sugars, starch or fructans, which constitute a buffer in case that source capacities are reduced as a result of drought-induced senescence. These reserves are readily utilized for grain filling, which may become a critical factor in sustaining grain filling when drought occurs during the peak of seed filling in wheat, rice, and barley (Yang and Zhang, 2006; Govind et al., 2011; Distelfeld et al., 2014; Zhang et al., 2015). Grain size determination is only initiated shortly after anthesis and during grain filling (Ji et al., 2010). Interestingly, with temperatures above 30°C assimilate transport from flag leaf to grain was substantially reduced but the stem transport was not affected even up to 50°C (Miyazaki et al., 2013). Stem reserves contribute ≥70% final grain mass (Rebetzke et al., 2008; Chochois et al., 2015; Zhang et al., 2015). Hence, breeding approaches should focus on increasing the stem sink potential to overcome heat and drought stress-induced yield and grain quality losses in cereals (Figure 1). A greater contribution of stem reserves play a critical role in maintaining yields under terminal drought stress. A wide genetic diversity in wheat stem WSC has been documented (Li et al., 2015a) and the introduction of the *Rht1* and *Rht2* dwarfing genes in wheat driving the green revolution is associated with a reduced WSC stem storage due to the shorter peduncles (Ellis et al., 2002; Wu et al., 2010), and the same could be the case with the introduction of semi dwarfing gene (*sd1*) in rice. Therefore plant height manipulation has been a crucial factor to readjust source-sink relationships and to improve yield stability.

Cereal crops store excess carbohydrates in the form of soluble sugars or sugar polymers within the vegetative tissues (Wehner et al., 2015). They are also capable of storing non-structural carbohydrates in the parenchyma cells of stems surrounding the vascular bundles located within internodes. Stem carbohydrates stored as sucrose, fructans (as in barley, wheat), or starch will be a good alternative source of assimilates when photosynthesis is impaired under post-anthesis stress (Joudi et al., 2012; Li et al., 2015a; Zhang et al., 2015). These studies suggested that one way to increase sink strength in the developing seed is through readjustment of non-structural carbohydrates in stems, which help to optimize carbon partitioning to increase kernel weight. Whole-plant carbon partitioning plays a vital role to buffer the source-sink interactions which may ultimately support yield stability by providing an alternative source when photosynthetic capacity is low during the period of drought stress. Accumulation of sugars in the stems may also help the plants to pull water from the soil into the vegetative parts of the plants through adjustment of turgor (Fu et al., 2011a). Such a readjustment is based on many interconnecting factors such as photosynthetic efficiency. Assimilate competition between organs (newly formed tillers, stem reserve accumulation versus seed biomass) and environmental influences such as water and nutrient availability, photoperiod and temperature. Genetic factors controlling assimilate partitioning eventually decide over seed filling.

Grain yield in cereals is a result of coordinated activities between source and sink tissues. Under optimal conditions, grain growth or seed yield are generally sink limited where as under stress treatments it will undergo source-limited sink dramatic readjustment. Therefore under terminal drought, yield losses in cereals are a result of both source and sink limitations. Yield reduction in barley and other crops even with adequate assimilates made available through artificial feeding to developing grains clearly highlights the importance of sink activity in determining yield under terminal drought (Boyer and Westgate, 2004). Sink strength plays a primary role in grain filling of cereals. But how pre-anthesis WSC reserves are related to the generation of sink strength especially under stress has not yet been explored in detail. Nitrogen (N) application at the spikelet differentiation stage improved pre-anthesis WSC reserves and sink strength in plants. Besides the lower number of endosperm cells being the limiting factor of sink strength, the rate of storage product accumulation and duration of seed filling has also been identified as another important stepping stone to increase grain weight under drought (Sreenivasulu et al., 2012).

As starch is the predominant storage form of carbohydrates in cereal grains, activities of enzymes involved in the conversion of sucrose to starch are major factors determining sink activity and hence crop yield (Sreenivasulu and Wobus, 2013; Wang et al., 2015). Among various enzymes involved in starch synthesis, sucrose synthase (SuSy), which catalyzes the conversion of sucrose to fructose and UDP-glucose is considered to be one of the important marker enzymes for sink strength in several crops including cereals (Worch et al., 2011; Hou et al., 2014). Its activity was found to be a major determinant of the duration of seed filling in barley and other cereals under both optimal and water-deficit conditions (Worch et al., 2011; Sreenivasulu and Wobus, 2013). On the other hand, reduction in the activity of acid invertase, another enzyme involved in the breakdown of sucrose especially during early stages of seed development in barley (Sreenivasulu et al., 2004) was pronouncedly inhibited under water limited conditions in wheat as well as in maize (Zinselmeier et al., 1995; Li et al., 2012a). Therefore, fine tuning of different sucrose cleavage pathways in a stage-dependent fashion is an important criterion for regulating seed metabolism under post-anthesis stress is essential.

ADP-glucose pyrophosphorylase (AGPase), an important rate-limiting enzyme of starch synthesis catalyzing the production of ADP-glucose was found to be negatively affected by severe drought stress in barley, wheat, and rice but moderate drying results show added advantage with increased rate of starch accumulation (Yang and Zhang, 2006; Seiler et al., 2011; Ruan, 2014; Wang et al., 2015). A notable exception to all the above results was reported in a controlled soil drying experiment carried out in rice and wheat during the grain filling (Yang and Zhang, 2006). These authors found that activities of SuSase, SSS, SBE (starch branching enzyme) and AGPase were significantly enhanced under moderate drought and were positively correlated with an increased rate of seed starch accumulation resulting in better seed weight compared to control but with reduced seed filling duration. Enhanced seed filling under mild drying was attributed to the accumulation of ABA which enhanced sink strength and remobilization of stem reserves (Seiler et al., 2011; Wang et al., 2015).

Starch rapidly accumulates in the central endosperm from early to mid grain filling and later at the periphery, whereas a shortage of assimilates during heat stress led to chalky rice grains due to lose packaging of amyloplasts (Wada et al., 2014; Sreenivasulu et al., 2015). Source-sink manipulation studies in rice have shown a close relationship between assimilate supply and white core chalk formation. With higher temperature a generally large-celled thick aleurone layer with irregular starch granules were formed leading to the trigger of chalk phenotype (Kim et al., 2012). Moreover, a 1°C increase above the optimal growing temperature of 25°C, the grain filling duration could be reduced by 2.8 days. Hence a line sufficiently equipped with stem reserves to overcome the reduced duration of grain filling induced with faster senescence induced either through heat stress or drought stress will be instrumental for matching optimum grain weight (Lobell et al., 2012; Reynolds et al., 2012).

## Regulatory Mechanisms Underlying the Initiation of Senescence and Nutrient Remobilization

### Hormonal Complexes Regulating the Initiation of Senescence

Among various phytohormones, ABA and CK are two major plant hormones having antagonistic effects on plant senescence under abiotic stress (Peleg and Blumwald, 2011). ABA mediated signaling cascade is known to promote senescence (Lee et al., 2015). Evidence has been presented for CK to inhibit leaf senescence, by expressing *IPT*, a key member of CK biosynthesis, under control of the senescence-associated genes *SAG12* or *SAG13*. This substantially delayed the initiation of senescence (Swartzberg et al., 2011). Further, it has been proved that CK inhibits senescence via an apoplastic invertase that produces extracellular hexoses. It appears that intracellular sugar sensing via hexokinase is dominant over extracellular sugar sensing with regard to leaf senescence (Swartzberg et al., 2011). *Arabidopsis* hexokinase (*AtHXK1*), an intracellular mitochondrial associated enzyme accelerates leaf senescence, while CK inhibits it (Cho et al., 2010). However, recent evidence suggested that apart from these two, there are also other hormones involved in a coordinated regulation of leaf senescence (Jaillais and Chory, 2010). In addition to their role in senescence, ABA and CK are also implicated in grain filling in different cereals, influencing endoreduplication, onset of seed storage and desiccation-related events (Sreenivasulu et al., 2010; Seiler et al., 2011) and thus seed yield (Tamaki et al., 2015). In a partial soil drying experiment during grain filling in wheat, the ABA content in the grain was found to positively correlate with enzymes involved in grain filling (Yang et al., 2003). ABA is also a well-known plant hormone which accumulates under stress and mediates transpirational loss through stomatal closure and thus ABA homeostasis is an important element in achieving water use efficiency (Seiler et al., 2014). Transgenic plants overexpressing 9-*cis*-epoxycarotenoid dioxygenase (*NCED*), an important enzyme in the ABA biosynthetic pathway under a drought-inducible promoter exhibited enhanced drought tolerance and maintained a better leaf water status and more green leaf area, whereas ubiquitous overexpression triggered senescence-related events (Thompson et al., 2000). Hence, for studying the role of hormones in plant development, it is necessary to use conditional promoters driving gene expression at a specific developmental stage or in response to specific environmental stimuli.

Ethylene is a gaseous plant hormone, associated primarily with fruit ripening, which promotes leaf senescence (Kim et al., 2015). The antisense suppression of 1-aminocyclopropane-1-carboxylic acid (ACC) oxidase, a key ethylene biosynthesis gene caused delayed leaf senescence (John et al., 1995). It is also known that ethylene alone may not be sufficient to initiate senescence and most likely that age-dependent factors are perhaps necessary for ethylene-regulated senescence. Characterizing mutants deficient in ethylene perception and signal transduction showed an enhanced leaf longevity in *ethylene-resistant 1* (*etr1-1*) and (*ein2/ore3*, Yang et al., 2008). Characterization of a large number of *onset of leaf death* (*old*) mutants confirmed the notion that the effect of ethylene on leaf senescence depends on age-related changes through *OLD* genes (Jing et al., 2005). These studies also proved that multiple genetic loci are required to regulate the action of ethylene in leaf senescence. In the *old1etr1* double mutant, in which ethylene perception was blocked, an age-dependent earlier onset of senescence occurred. Altogether, these experiments suggest that *OLD1* negatively regulates the integration of ethylene signaling into leaf senescence. Ethylene-induced *SAGs* together with physiological studies revealed extensive cross talks between ethylene and other hormones that are associated with the progression of leaf senescence.

### Transcription Factor Cascades Regulating Senescence

Genetic analysis revealed that senescence is controlled by *senescence associated genes (SAG)* acting in the loop as various negative and positive regulators (Gregersen et al., 2008; Masclaux-Daubresse et al., 2014; Huang et al., 2015). However, the complete transcription factor and signaling cascades involved in regulating leaf senescence are still unknown. Notably, several regulatory elements, such as signal transduction-related proteins and transcription factors were identified among the SAGs in cereals (Hollmann et al., 2014), and some of their functions were validated utilizing *Arabidopsis* T-DNA insertion lines (Lim et al., 2007). Interesting observations revealed that the transcription factors *WRKY53* and *AtNAP* (a member of NAC TFs family) act as positive regulators of leaf senescence coordinating the progression through the final stages of leaf development (Guo and Gan, 2006; Ay et al., 2009). The importance of the senescence-induced remobilization of nitrogen in crop plants is exemplified by the map-based cloning of the grain protein concentration (*GPC*) locus, *NAM-B1* (encoding a NAC transcription factor) originally identified in wheat chromosome 6B. The presence of a functional *NAC* gene was found to increase the grain protein content as a result of an earlier induction of post-anthesis senescence (Uauy et al., 2006). A similar gene was also identified on chromosome 6H of barley (*HvNAM-1*) through QTL analysis, that explained 45% of the heritable variance in protein content of a mapping population (Jamar et al., 2010). Recently, using near isogenic lines developed for the 6H locus, it was found that, in addition to acceleration of post-anthesis flag leaf senescence, the *GPC* locus also accelerated the pre-anthesis development from transition of the shoot apical meristem (SAM) onwards (Jukanti and Fischer, 2008; Parrott et al., 2012). Transgenic wheat lines, in which expression of *NAM-B1* and its homeologous genes were down-regulated using RNAi, were characterized by delayed leaf senescence and lower grain protein, Fe and Zn concentrations (Waters et al., 2009). The authors concluded that wild wheat encodes a NAC transcription factor (*NAM-B1*) which accelerates senescence along with the remobilization of protein and nutrients such as iron and zinc from leaves to grains. Transgenics overexpressing *NAP* showed premature senescence. Moreover, a *NAP* homolog of rice restored delayed leaf senescence in the *AtNAP*-deficient *Arabidopsis* mutant (Guo and Gan, 2006).

The *WRKY* family (zinc finger type) of TFs is another important family (the second largest group of TFs) associated with senescence as well as disease resistance (Zentgraf et al., 2010). In dark-induced senescence, 21 *WRKY* TFs out of the 59 known WRKYs were differentially expressed (Friedel et al., 2012; Li et al., 2012b). However, the function of the individual WRKY factors that are expressed during senescence is not yet very clear. Several reports pointed out that WRKY factors act in a regulatory network, in which the transcription of other WRKY factors is influenced, rather than in a linear signal transduction pathway. *WRKY53* has been identified in *Arabidopsis* as an important factor in controlling leaf senescence (Zentgraf et al., 2010). Inhibiting the function of *WRKY53* using RNA interference retarded leaf senescence (Miao et al., 2004). These authors have identified more than 60 targets of WRKY53 including six other members of the WRKY gene family. It appears that WRKYs act as an upstream element in the signaling pathway. Though WRKY TFs have been shown to be involved in the regulation of leaf senescence, not much is known about the upstream regulation of the senescence-specific expression of *WRKY* factors. DNA-binding protein with an unknown function that contains a transcriptional activation domain and a kinase domain regulates WRKY53 transcription factor (Miao et al., 2008). *In vitro* studies revealed that this activation domain protein (AD protein) can phosphorylate itself, and that phosphorylation increased its DNA-binding activity to the WRKY53 promoter region. Moreover, the AD protein interacted with a mitogen-activated protein kinase kinase 1 (MEKK1). These studies revealed that that there may be competition between WRKY53 and the AD protein for binding of *MEKK1* at the *WRKY53* promoter, and that the AD protein is a positive regulator of *WRKY53* expression. The transcriptional factor GATA4 and an orthologous gene product S40-3 have been reported to regulate the expression of *WRKY53* and positively regulate the senescence process (Fischer-Kilbienski et al., 2010). The *s40-3a* mutant, carrying a T-DNA insertion in the promoter region, exhibited a stay-green phenotype. Thus, both GATA4 and S40-3 are supposed to play a significant role in senescence regulation. Besides the central role of WRKY53 in senescence regulation, WRKY6 and WRKY22 have been reported as positive regulators of senescence (Lim et al., 2007).

Kinases that are involved in senescence regulation by hormones are members of the ORE (oresara) family, a member ORE12-AHK3 is a histidine kinase-type CK receptor (Kim et al., 2006). AHK3 is mainly involved in CK signaling, as a loss-of-function *ahk3* mutant displayed an early senescence phenotype. As discussed above, sugars exert a regulatory influence over senescence. Certain protein kinases sense the sugar level and regulate sugar-mediated signaling. One such protein kinase is SnRK1 (Snf1-Related Kinase), which acts as a translational inhibitor and a transcriptional inducer for a wide range of proteins or genes influencing development and environmental responses (Robaglia et al., 2012). SnRK-1 can be activated by darkness, nutrient starvation and high sucrose or low glucose concentrations in the cell. Down-regulation of *SnRK1* led to a number of developmental irregularities including premature senescence. Extracellular invertase, by hydrolyzing sucrose to hexoses, counteracts the influence of SnRK1. Other sugar-sensing senescence regulators include hexose kinase (Lee et al., 2015). In *Arabidopsis*, several HXKs act as sensors of the glucose level and are associated with mitochondria. However, other HXKs can also be found in the nucleus in higher-molecular weight complexes, which repress the expression of photosynthetic genes. The *Arabidopsis gin2-1* mutant carries a non-sense mutation in the *HXK1* gene exhibited delayed senescence due to alteration in glucose sensing. Moreover, this mutant showed a reduced senescence response to glucose feeding and reduced sugar accumulation (Pourtau et al., 2006). These experiments indicated that senescence is associated with *HXK1*-dependent signaling.

## Conclusion

Through empirical means physiologists have identified “stay-green”—defined by the extended lifespan of photosynthetic activity under challenging environments, as an antagonist to senescence—defined by the breakdown of chlorophyll. Crop yield under water deficit or heat stress strongly depends on photosynthates provided either through current photosynthesis or through the remobilization of stored carbohydrates from the stem. An alternative assimilate source to reduced photosynthesis under drought stress consists of carbohydrates (sugar, starch, and fructans) that were built up during pre-anthesis and stored in the stem. These reserves may be utilized during grain filling, especially if current photosynthesis is reduced due to drought in cereals. However, this mechanism is effective only during seed filling, but not necessarily advantageous during the critical stages encompassing gametogenesis, anthesis or fertilization (–10 days before fertilization until 8 days after fertilization). The relative importance of these two plant strategies (stay-green versus remobilization) for efficient seed set and seed filling under terminal drought depends on genotype plasticity and its ability to cope with the severity of stress. Thus, what underpins the crop productivity ultimately under drought stress therefore depends on how the crop (i) captures assimilates to maintain pollen viability and safeguard fertilization and (ii) undertakes effective assimilate partitioning to sink organs.

Several studies have proposed to use the starch content per seed as a measure for a drought tolerance index, but other studies have demonstrated an increase in seed number with only a marginal impact on seed weight. Thus, there is a need to dissect how stay-green and remobilization phenotypes impact on yield stability by influencing grain number and grain weight, as both components determine yield stability. Occurrence of drought stress during gametogenesis, anthesis and onset of seed development is critical, resulting in impaired grain set, reduced grain weight and yield loss. This is thought to be at least partly due to a decrease in photosynthetic efficiency and to changes in sucrose cleavage processes in reproductive organs. Although invertases and their activity are negatively affected by stress and further deteriorated under elevated ABA levels, resilient sources of invertases have lost attention. The recent identification of such resilient invertases and sucrose synthase in wheat supports molecular marker development and promises to overcome the identified bottle-neck in sugar conversion to facilitate an undisturbed supply of sugars to the sensitive reproductive organs. Assessing the population for yield stability under heat, drought and combined drought and heat stress with defined stable QTLs for yield from wheat mapping populations in field trials help to develop climate resilient varieties through breeding (Bennett et al., 2012; Bonneau et al., 2013). The switch between stay-greenness and senescence and its influence on seed yield stability is still elusive. Several studies employing the NDVI (normalized difference vegetation index) technique, stay-green during physiological maturity and rate of senescence were shown to positively and negatively correlate with yield, respectively, under terminal stresses. Hence, the use of a high-throughput chlorophyll fluorescence or alternative techniques with more specific spectral indices to estimate the senescence pattern and to identify the elusive switch from stay-green to senescence, is needed. With the unraveled molecular mechanisms it is possible to breed for functional stay-greenness in cereals by optimizing assimilates, modify photorespiration and enhance sink strength to attain yield stability by defining distinct strategies during anthesis under post-anthesis abiotic stresses. Moreover, drought-tolerant wheat germplasm that is able to maintain source strength when stressed at the young microspore stage failed to maintain sink strength when stressed at anthesis and grain-filling (Ji et al., 2010). This provides an excellent example of a pre-anthesis “stay-green” phenotype and for an enhanced remobilization efficiency phenotype that operates independently (Figure 1). Hence, we propose future cereal stress breeding programs to exploit these unique phenomena by identifying lines or accessions possessing these features. Such contrasting lines may subsequently be used to develop lines with a pre-anthesis stay-green and a post-anthesis remobilization phenotype.

## Author Contributions

Overall concept of the review has been designed and written by NS. All other authors of the manuscript meet the essential criteria of the publication by contributing to individual sections and figure. All authors have read and approved the manuscript.

### Conflict of Interest Statement

The authors declare that the research was conducted in the absence of any commercial or financial relationships that could be construed as a potential conflict of interest.
